# Editorial: Establishing and Maintaining Cell Polarity

**DOI:** 10.3389/fcell.2021.722003

**Published:** 2021-06-30

**Authors:** Benjamin Lin, Zhiyi Lv, Yi Wu

**Affiliations:** ^1^Department of Cell Biology, Kimmel Center for Biology and Medicine of the Skirball Institute, New York University School of Medicine, New York, NY, United States; ^2^Sars-Fang Centre, Institute of Evolution and Marine Biodiversity, Ocean University of China, Qingdao, China; ^3^Department of Cell Biology, R.D. Berlin Center for Cell Analysis and Modeling, UConn Health, Farmington, CT, United States

**Keywords:** mechanotransduction, polarity (cell), signaling/signaling pathways, development, multi-cue integration

Cell polarity arises from compartmentalizing molecular effectors within the membrane, cortex, and/or cytoplasm. These asymmetric molecular ensembles serve as templates for signaling cascades which drive essential cell functions, including cell division and migration, and are coordinated across the tissue scale during morphogenesis and barrier formation. Polarity is initiated through symmetry breaking via an internal or external cue and is typically maintained through antagonistic interactions amongst opposing effectors. At present, many of the molecular components involved in generating polarity have been identified, in large part through genetic screens in model organisms. Furthermore, an ever expanding palette of genetically encoded fluorescent proteins and biosensors coupled with continued advances in microscopy have made observing polarity extremely tractable. This has led to an expansion of quantitative imaging which in turn has fueled the development of mathematical models, which both describe and predict new properties of polarity systems. Despite this progress, the complex interplay between core polarity modules, the various external cues which influence them and the cytoskeleton are still being unraveled in pursuit of understanding how polarity is disrupted in disease.

In this Research Topic, one of our aims was to provide a venue for members of the polarity field to review its current state and propose future directions. Given the ubiquity of polarity in development, we have accordingly received contributions from the perspective of several model organisms, including *Drosophila, C. Elegans, Ciona*, and *S. cerevisiae*. We also highlighted specific areas of interest, including how cells might resolve multiple cues, how polarity can be reconstituted in apolar cells, and how mechanical and chemical cues are integrated. We were delighted to not only receive original research, reviews, and methods articles which explore these questions but other underrepresented areas as well. Below we provide a brief synopsis of the articles in this collection and encourage readers to delve further into each one.

Polarity is initiated through symmetry breaking via the amplification of a local asymmetry through positive feedback. Local asymmetries can arise spontaneously or can be induced by a cue. Gan and Motegi provide a comprehensive review of the interplay between mechanical forces and chemical signaling during symmetry breaking in the *C. Elegans* zygote. Along with membrane and cortical polarity, cytoplasmic polarity also contributes to important cell functions, including the specification of soma and germline. Kim and Griffin review the role of Polo-like kinase 1 (PLK-1) in the first asymmetric division of the *C. Elegans* embryo, noting how cytoplasmic gradients of PLK-1 play pleiotropic roles in establishing cortical domains and regulating the correct segregation of fate determinants to the germline.

The two contributions above exemplify the complexities arising when studying polarity. In particular, teasing apart how different polarity modules influence each other is not trivial when the networks are interconnected. Johnston describes a reductionist technique to reconstitute polarity in apolar cells which has been successfully utilized to assess the sufficiency of different proteins to orient cell division. Controlling the cell division axis is particularly crucial in blood vessel development, where divisions parallel to the vessel can elongate it while perpendicular divisions can expand the diameter of the existing vessel or support sprouting of new vessels. Wu et al. review how mitotic spindle polarity is oriented in blood vessel development, highlighting the roles of electric currents and vascular endothelial growth factor (VEGF) in this process.

Individual cell polarity can be coordinated across cell collectives during morphogenesis via mechanotransduction through trans-interacting junctional complexes connected to the cortex. Canonical examples include ratcheted apical constrictions driving tissue involution and folding during gastrulation and planar polarized forces producing tissue elongation through convergent extension. Kong and Großhans review E-cadherin mechanotransduction in two contexts- (1) planar polarity dependent convergent extension in *Drosophila* embryonic epidermal cells and (2) interactions between two epithelial populations during tissue closure. The importance of polarity coordination between multiple cell populations is also apparent in an original research contribution from Kunz et al. which reveals that apical constriction and dendritic towing act to correctly position sensory organs in a proteasome dependent manner. Mechanical forces from polarized extracellular matrix (ECM) and supracellular cytoskeletal structures can also influence tissue morphogenesis. Popkova et al. review how planar polarity movement aligned ECM and basal supracellular actin stress fiber contractions act to sequentially elongate *Drosophila* eggs along the anterior-posterior axis through constraining isotropic growth. Although planar cell polarity (PCP) and apical-basal (AB) polarity can act in isolation in some contexts, interactions between these two polarity systems can be critical for tissue morphogenesis. Peng et al. review the interplay between PCP and AB polarity during notochord formation in *Ciona* as notochord cells transition from a planar sheet to an ECM wrapped tube with a hollow lumen.

Polarity can be influenced by a variety of cues, including electric fields, shear stress, cell contact, and chemical ligands. How does the presence of one cue modify the response to another and is there a context dependent hierarchy between them? Tardy et al. utilize *Drosophila* embryonic hemocytes (macrophage equivalent in *Drosophila*) to provide some insight into these questions. They uncover how Spitz [a *Drosophila* epidermal growth factor (EGF) ligand] signaling from ectopic sources can compete and dampen hemocyote responses toward both developmental migratory cues, such as apoptotic cells, and acute migratory wound cues.

Lastly, an intriguing question is how existing polarities are remodeled. Epithelial cells can transiently redistribute both apical and basolateral polarity proteins during cell division and re-establish polarity afterwards. Similarly, bud growth in *Saccharomyces cerevisiae* transitions from apical to isotropic by removing polarized Cdc42 activity at the bud tip via redistribution of its activator. Quadri et al. propose a general role for Ras in resolving polarity and highlight its role downstream of a kinase, Haspin, in binding and changing the localization of a Cdc42 activator to prevent hyperpolarization during mitosis. In another contribution from this group (Galli et al.), Haspin is shown to be involved in enforcing the morphogenesis checkpoint, which halts the cell cycle until proper polarity is established.

We would like to thank the authors and reviewers for their efforts in compiling this impressive set of articles in this Research Topic which provide a bird's eye view of the polarity field through the lens of model organisms and addresses underrepresented aspects of polarity, such as multi-cue interpretation, resolution, and reconstitution. We hope this collection provides an impetus for further work to ultimately understand how polarity is dysregulated in disease.

## Author Contributions

BL wrote the manuscript and received input from ZL and YW. All authors contributed to the article and approved the submitted version.

## Conflict of Interest

The authors declare that the research was conducted in the absence of any commercial or financial relationships that could be construed as a potential conflict of interest.

